# DIG-DUBs: mechanisms and functions of ISG15 deconjugation by human and viral cross-reactive ubiquitin proteases

**DOI:** 10.1042/BST20240859

**Published:** 2025-07-09

**Authors:** Thomas Bonacci, Michael J. Emanuele

**Affiliations:** Department of Pharmacology, Lineberger Comprehensive Cancer Center ,The University of North Carolina at Chapel Hill, Chapel Hill NC 27599, U.S.A.

**Keywords:** DUB, ISG15, ubiquitin, USP18

## Abstract

Interferon-stimulated gene 15 (ISG15) is a ubiquitin-like protein and, as such, acts as a post-translational modifier that plays a critical role during bacterial and viral infections after interferon (IFN) production. As part of the innate immune system, ISG15 is strongly induced by type I IFNs, and its conjugation to intracellular proteins and viral proteins (ISGylation) allows cells to fight off infection. Importantly, ISGylation is a reversible process that is largely mediated by the cysteine protease USP18 (Ubp43 in mice). As a multifaceted protein, USP18 is a major negative regulator of IFN signaling and the predominant deISGylating enzyme in humans. However, in recent years, additional proteases such as USP16 and USP24 have been reported to also mediate ISG15 hydrolysis. Moreover, coronaviruses and other viral pathogens often encode proteases that possess deISGylating activity, which promotes viral infection by antagonizing ISGylation. Here, we review various enzymes and modes of action employed by human and viral proteases to regulate deISGylation under physiological or pathogenic conditions.

## Post-translational modifications by ubiquitin and ubiquitin-like proteins

**Figure 1:**
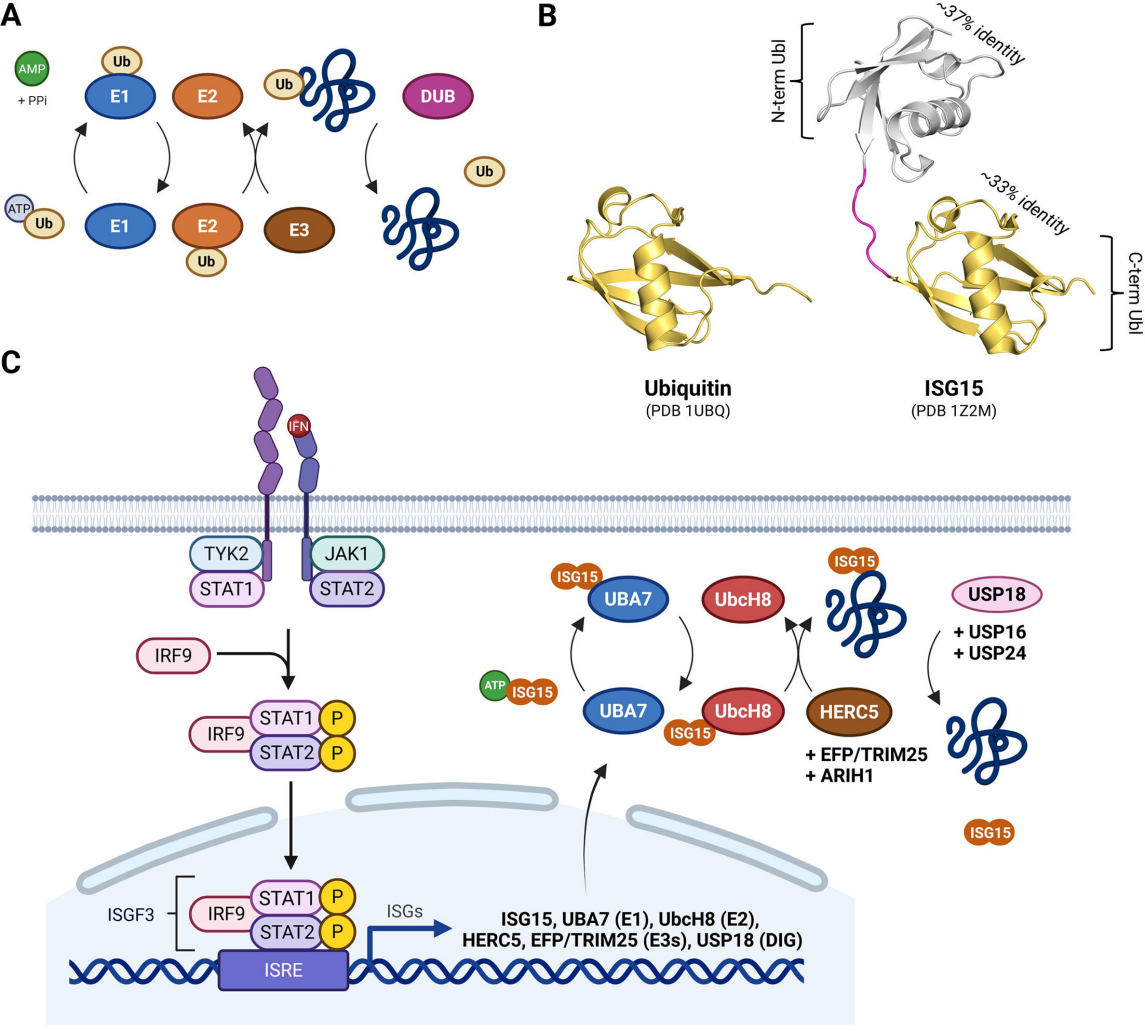
The Ub and ISG15 conjugation pathways. (**A**) The ubiquitin conjugation machinery relies on ubiquitin-activating enzymes (E1, 2 in humans), ubiquitin-conjugating enzymes (E2, ~40 in humans), and ubiquitin ligases (E3, >600 in humans), which leads to the covalent modification of a substrate by ubiquitin. Deubiquitinating enzymes (DUBs, ~100 in humans) cleave the isopeptide bond between the C-terminal carboxyl group of ubiquitin and a side-chain amine group of a lysine residue on the substrate. (**B**) Crystal structures of ubiquitin (PDB 1UBQ) and ISG15 (PDB 1Z2M), with the percentage of sequence identity between Ub and each ISG15 Ubl domain. The hinge region, corresponding to residues 76–82 in humans (DKCDEP), is colored in magenta. (**C**) Type I interferon is recognized by the heterodimeric receptor made of the subunits IFNAR1 and IFNAR2, which associates with the Janus activated kinases (JAKs), tyrosine kinase 2 (TYK2), and JAK1, respectively. JAKs activation phosphorylate signal transducer and activator of transcription 2 (STAT2) and STAT1, which in turn leads to the formation of STAT1–STAT2–IRF9 (IFN-regulatory factor 9) complex. This complex then translocates to the nucleus and binds IFN-stimulated response elements (ISREs) in DNA to initiate gene transcription of hundreds of interferon-stimulated genes (ISGs), which include, ISG15, UBA7 (E1), UbcH8 (E2), HERC5 and EFP/TRIM25 (E3s) and USP18 (DIG). Only USP18, USP16, and USP24 are shown, since they have been tied to substrates. DIG, deISGylating enzyme. Created with BioRender.com.

Post-translational modifications (PTMs) by members of the ubiquitin-like (Ubl) family of proteins are involved in the regulation of virtually every physiological process [1]. The founding member of this family, ubiquitin, gets conjugated to proteins through a three-step enzymatic cascade involving the sequential action of a ubiquitin-activating enzyme (E1), a ubiquitin-conjugating enzyme (E2), and a ubiquitin ligase (E3) to covalently attach ubiquitin onto a lysine residue on the target protein [2] (Figure 1A), although ubiquitination of serine, threonine, and cysteine residues, as well as non-protein substrates, has been described [3–5]. Ubiquitin can be added as a monomer onto a single lysine residue (mono-ubiquitination), several lysine residues (multi-ubiquitination), or form chains (poly-ubiquitination) by adding additional ubiquitin molecules to the first one [6]. The most well-known function of ubiquitination is to target proteins for degradation by the 26S proteasome, which is referred to as the ubiquitin-proteasome system [7,8]. Decades of research have shown that ubiquitination has a plethora of important signaling functions beyond proteolysis, and these include the regulation of protein–protein interactions, endocytosis, checkpoint signaling, protein function, and localization [1]. The variety of outcomes following ubiquitination stems from the diversity of ubiquitin conjugation types [9], as well as the highly complex nature of the enzymatic cascade, which is illustrated by the hundreds of genes involved in the process (Figure 1A).

## Deubiquitinating enzymes: antagonists of ubiquitination

Like other PTMs, ubiquitination is reversed by proteases called deubiquitinating enzymes (DUBs), and ~100 are encoded in the human genome [10]. DUBs hydrolyze the isopeptide bond between the C-terminal carboxyl group of ubiquitin and a side-chain amine group of a lysine residue in the substrate protein [11]. DUBs antagonize ubiquitination (Figure 1A) and regulate diverse cellular pathways [12], since they maintain the balance between conjugation/deconjugation. Interestingly, among the ~500 genes encoding proteases in our genome, DUBs account for ~20%, making them the largest family of proteases in humans [13]. Based on their enzymatic activity, DUBs can be classified into two main categories, i.e. cysteine proteases or metalloproteases [11]. The cysteine-based DUBs include six families, each with their own catalytic domain (CD): ubiquitin-specific proteases (USPs), ovarian tumor proteases (OTUs), ubiquitin carboxy-terminal hydrolases (UCHs), Machado–Joseph disease proteases (MJDs), motif interacting with ubiquitin-containing novel DUB family (MINDY), and zinc finger with UFM1-specific peptidase (ZUFSP). The metalloprotease DUBs include only the zinc-dependent JAB1/MPN/MOV34 metalloproteases (JAMMs) family.

## The Ubl ISG15 and its antiviral role

In humans, 16 Ubls exist, and they all share sequence and structural similarity with ubiquitin (Figure 1B). Ubls are also conjugated onto lysine residues by similar enzymatic cascades as ubiquitin, albeit using their own E1/E2/E3 conjugation machinery [14]. The first Ubls described were interferon-stimulated gene 15 (ISG15) [15,16], neural precursor cell expressed, developmentally down-regulated 8 (Nedd8) [17,18], and the small ubiquitin-like modifier (SUMO) family of proteins [19,20]. In contrast to ubiquitin, Nedd8, and SUMO, ISG15 structurally resembles two Ubl molecules which are connected by a short linker, or hinge region [21,22] (Figure 1B). Moreover, whereas ubiquitin, Nedd8, and SUMO are constitutively expressed in cells, ISG15 is strongly induced by type I interferons (IFN-α and IFN-β) [23] in response to infections (viral, bacterial, and parasite) [24,25], polyinosinic:polycytidylic acid (poly I:C) [26], or lipopolysaccharide [27]. Mechanistically, interferon activates the janus activated kinases (JAK)/signal transducer and activator of transcription (STAT) signaling pathway, triggering the transcription of hundreds of ISGs [28] (Figure 1C). Among them, ISG15 is translated as a 17-kDa precursor that is processed into its conjugatable 15-kDa form, when its carboxy-terminal sequence LRLRGG necessary for its covalent attachment to target proteins becomes exposed [15]. The canonical ISG15 conjugation machinery includes E1 UBE1L/UBA7 [24,29], E2 Ube2L6/UbcH8 [30,31], and E3 ligase HERC5 [32,33] (Figure 1C). In addition to ISG15 itself, the expression of this core conjugation machinery is also triggered by interferon, which explains the massive increase in protein ISGylation following infection. Two additional E3s, Ariadne homologue 1 (ARIH1) and estrogen-responsive finger protein [EFP; also known as tripartite motif (TRIM25)], have also been reported [34–37]. ARIH1 is an E3 from the RING-in-between-RING family [38], which was recently found to act as an E3 ISG15 ligase toward cGAS [34], the critical sensor of viral DNA in the cytoplasm [39]. ARIH1 mono-ISGylates cGAS on lysine 187, which induces its oligomerization, thereby promoting antiviral immunity and autoimmunity [34]. EFP is an E3 which regulates the innate immune response against viruses [40]. EFP is a member of the TRIM family of proteins, which are characterized by a TRIM on their N-terminus containing a RING domain, one or two B-box domains, and a coiled-coil region [41]. Like the canonical ISG15 conjugation machinery, the expression of EFP and other TRIM proteins is induced by both type I and II interferons [42], and it was shown that EFP can ISGylate 14-3-3σ [35], even though the function of this modification still remains unknown. Intriguingly, EFP has also been shown to act as an E3 ISG15 ligase for Proliferating Cell Nuclear Antigen (PCNA) and p53 in the context of DNA damage [36,37]. Finally, it has also been reported that the RING domain of EFP mediates its auto-ISGylation on lysine 117, which negatively regulates its ISG15 E3 ligase activity toward 14-3-3σ [43]. Despite these reports, it is believed that HERC5 is the dominant E3 ligase for ISGylation in human cells, since little, if any, conjugates can be observed in its absence [32,33]. Finally, while ISG15 can be found inside cells and conjugated to intracellular substrates, it can also exist as a free, unconjugated, extracellular molecule, where it acts as a cytokine [44]. The conjugation-independent functions of ISG15 are beyond the scope of this article, but we point interested readers to excellent reviews regarding ISG15 function as a signaling molecule [45–47].

**Figure 2:**
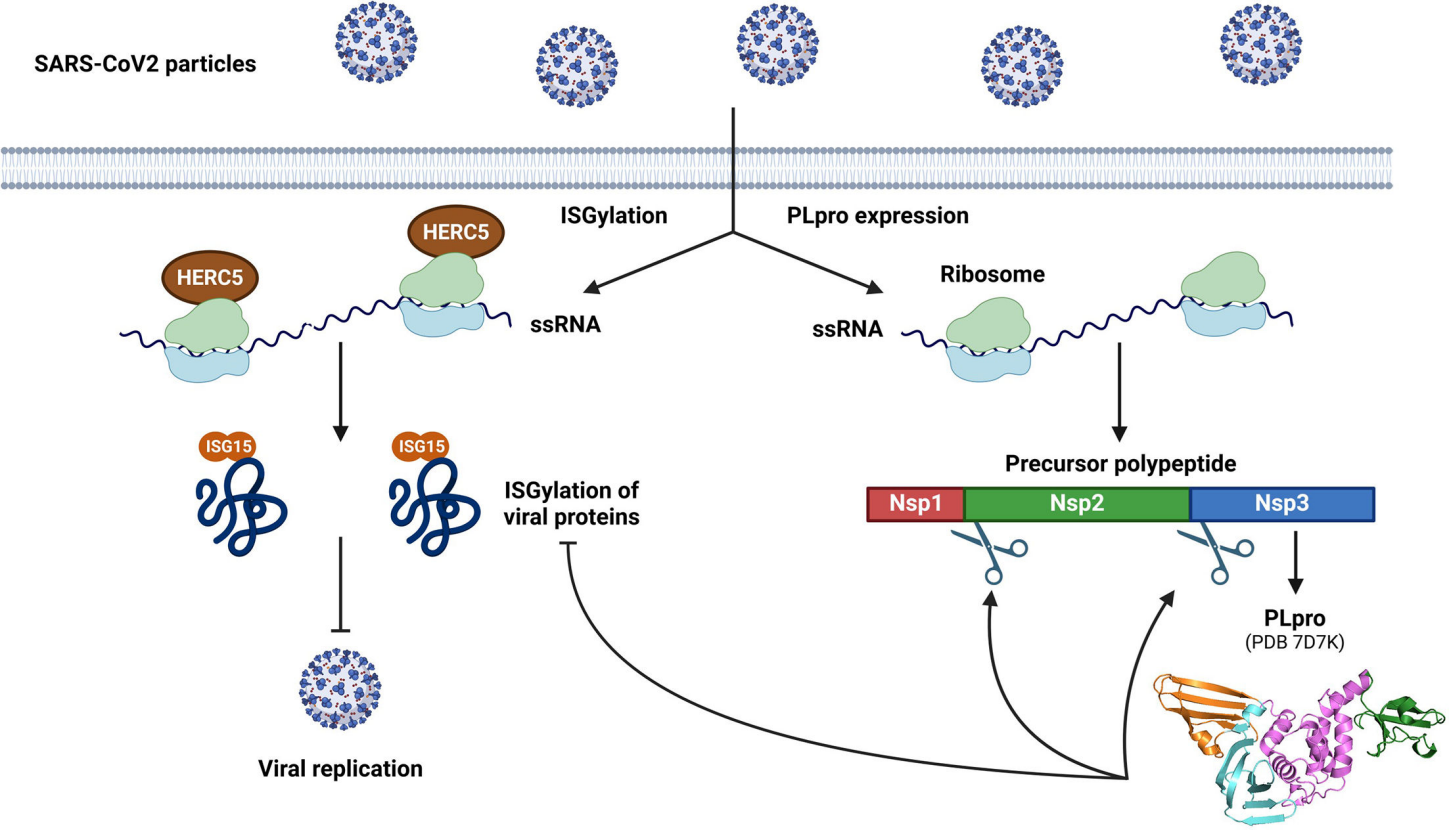
Selected example of ISG15 cellular function and its hijacking by PLpro from SARS-CoV-2 coronavirus. Following infection and interferon production, the ISG15 E3 HERC5 associates with polysomes, which leads to ISGylation of structural viral proteins that are being synthesized to ultimately inhibit virus replication. In parallel, the DIG/DUB PLpro (PDB 7D7K) from SARS-CoV-2 processes the precursor polypeptide pp1a and hijacks ISGylation of structural viral proteins, thereby facilitating virion assembly and replication. The domains of PLpro^CoV2^ include the fingers (orange), palm (cyan), thumb (violet), and Ubl (green). Created with BioRender.com.

ISGylation differs from ubiquitination in several ways, since ISG15 is conjugated as a monomer and ISGylation is not a proteolytic signal. Notably, it has been described that ISG15 can form mixed chains with ubiquitin [48], even though the physiological consequence of such polymers remains elusive. Instead, as part of the innate immune system, ISGylation contributes to the host response following bacterial and viral infections [46]. This is achieved through co-translational attachment of ISG15 to viral capsid proteins, as well as host cellular proteins, which ultimately inhibits virus assembly and/or replication [49]. This function stems from the observation that the bulk of ISGylation occurs on proteins that are being synthesized, which was attributed to co-localization of the ISG15 E3 HERC5 with polysomes (Figure 2, left). The rationale is that immediately after infection, a significant portion of newly made proteins are of viral origins, and the spatial localization of the ISG15 machinery at sites of protein translation would guarantee ISGylation of these viral effectors. The importance of ISG15 in coordinating the host immune response is illustrated by the fact that it is one of the most highly up-regulated proteins during viral pathogenesis [28]. Accordingly, viruses from diverse families have evolved strategies to antagonize ISGylation to favor virulence (Figure 2, right). Furthermore, genetic studies from ISG15-deficient patients, ISG15^−/−^, and UBE1L^−/−^ animal models, as well as cellular models, all point out to a role in stimulating the immune system [50–52]. Importantly, the outcome of ISGylation depends on factors such as the type of pathogens (viruses, bacteria), the host (mouse or human), the ratio of conjugated to free ISG15, or the cellular location of unconjugated ISG15 (cytosol or secreted). Finally, despite its clear role as an antiviral agent [46], ISG15 has also been linked to autoinflammatory interferonopathies [53–55], the homeostasis of the central nervous system [56], autophagy [57], cancer [58], and the DNA damage response [59].

## Reversibility of ISG15 conjugation by human proteases

**Figure 3:**
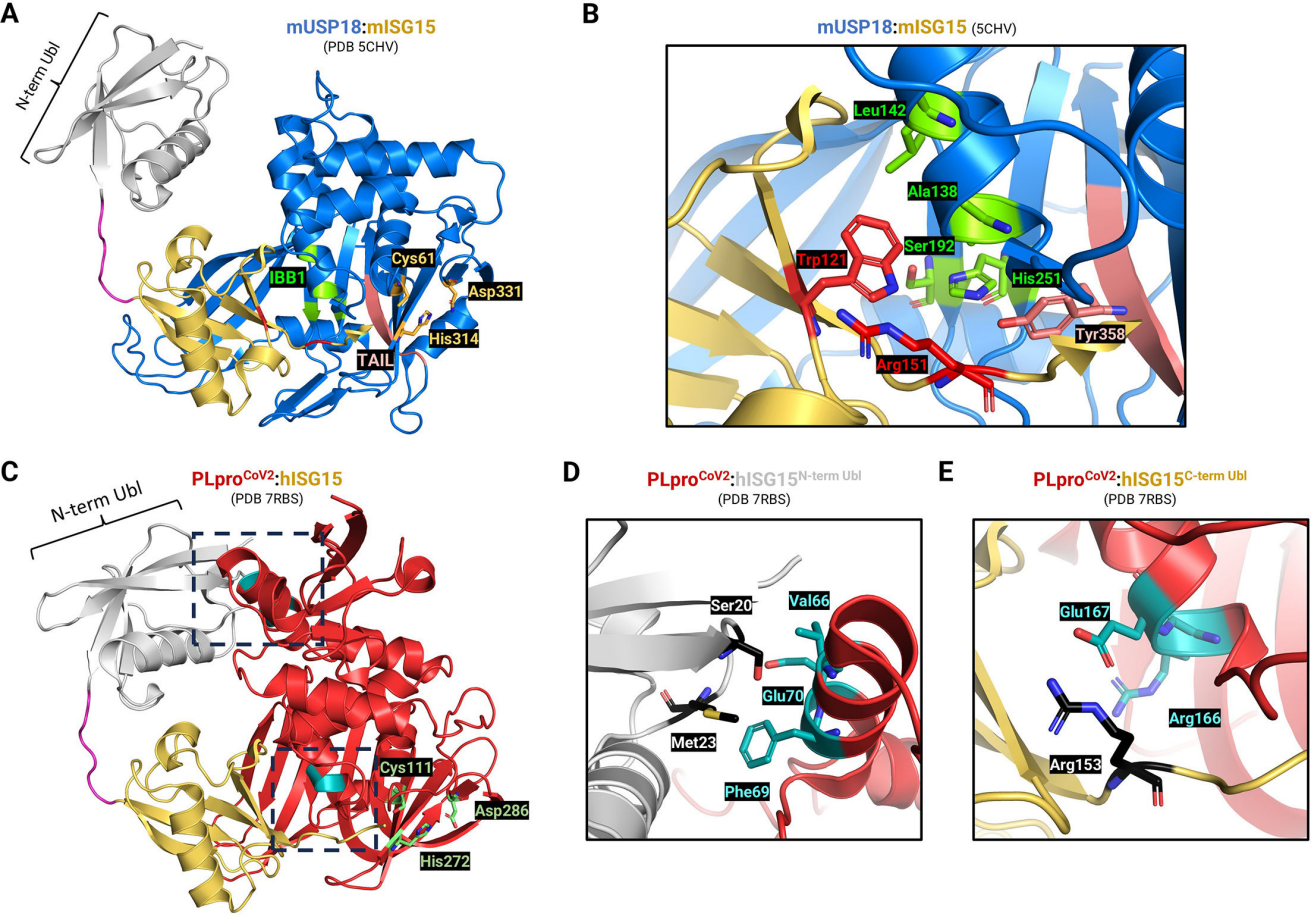
Different mechanisms of ISG15 recognition by USP18 and PLpro^CoV2^. (**A**) Structure of the catalytic domain of mUSP18/Ubp43 in complex with mouse ISG15/mISG15 (PDB 5CHV). mUSP18/Ubp43 catalytic triad (Cys61, His314, Asp331) is shown in gold, the ISG15-binding box 1 (IBB1) is in green, the TAIL motif is in salmon, and mISG15 hinge region, corresponding to residues 74–80 (QNCSEP), is colored in magenta. (**B**) Close-up view of the residues involved in the interaction between mUSP18/Ubp43 and mISG15. The IBB1 residues of mUSP18/Ubp43 (Ala138, Leu142, Ser192 and His251) mediate interaction through Trp121 of mISG15, while Tyr358 from the TAIL motif likely engages Arg151 of mISG15. (**C**) Structure of PLpro from SARS-CoV-2 in complex with human ISG15/hISG15 (PDB 7RBS). PLpro^CoV2^ catalytic triad (Cys111, His272, Asp286) is shown in lime green. Dashed boxes indicate interacting regions between hISG15 and PLpro^CoV2^ residues, which are in teal color. (**D**) Close-up view of the upper dashed box from (**C**) showing Ser20 and Met23 from hISG15 N-term Ubl (black residues) interacting with Val66, Phe69, and Glu70 on PLpro^CoV2^ (teal residues). (**E**) Close-up view of the lower dashed box from (**C**) showing Arg153 from hISG15 C-term Ubl (black residue) interacting with Arg166, and Glu167 on PLpro^CoV2^ (teal residues).

ISG15 is removed from substrates by proteases called deISGylating enzymes (DIGs) named by analogy to their DUB counterparts, but underlining their specific ability to deconjugate ISG15 [60]. Protein deISGylation is largely mediated by USP18, which was cloned by the Zhang lab from AML1-ETO knock-in mice [61,62]. Based on the protein sequence, this gene belongs to the USP family of DUBs, and deubiquitinating activity was supported by initial biochemical assays. However, shortly after its cloning, the Zhang lab evaluated the potential role for USP18 as a DIG [63]. *In vitro* enzymatic assays showed that USP18 exhibits activity for ISG15, but not for Ub, Nedd8, or SUMO1, indicating that USP18 was a DIG, rather than a DUB. Furthermore, deletion of the mouse ortholog of USP18 (Ubp43) by homologous recombination led to a massive increase in protein ISGylation [63], leading to the conclusion that USP18 is the major DIG in mammals. Because of its deISGylating activity, USP18 acts as an antiviral agent, but its function has also been linked to autoimmune diseases, such as interferonopathies [64,65] and cancer [66–69]. However, it is important to note that the general belief of USP18 being exclusively a DIG has remained a conundrum, since several studies clearly support the notion that it can also act as a DUB in some cases [70–76]. Whether USP18 is a DIG or a DIG/DUB, its exceptional ability to deconjugate ISG15 from virtually any ISGylated protein is unique among these types of proteases. This molecular characteristic has been the focus of two studies which aimed at explaining the basis of this enzymatic property [77–79]. By solving the crystal structure of Ubp43 CD in complex with mouse ISG15 (mISG15), Basters et al. revealed that mUSP18/Ubp43 recognizes ISG15’s C-terminal Ubl domain, leaving the N-terminal Ubl domain available for additional interactions [78] (Figure 3A). Moreover, mISG15 harbors a hydrophobic patch on its C-terminal Ubl domain centered around Trp121 (Trp123 in human), which is accommodated by a shallow pocket on the surface of mUSP18/Ubp43 (Figure 3B). This surface was named ISG15-binding box 1 (IBB-1) and comprises four residues (Ala138, Leu142, Ser192, and His251, Figure 3B) crucial for catalysis, since mutating them strongly impaired the interaction between mUSP18/Ubp43 and mISG15.

We also recently uncovered new features of USP18 enzymatic activity [80]. To characterize new molecular determinants of USP18 toward ISG15, we performed a comparative analysis with USP18’s human paralog, USP41. Of note, USP41 has been recently annotated as a pseudogene after it was reported to lack both 5′ and 3′ UTR regions [81]. However, USP41 transcripts were found by RNA-sequencing among the top up-regulated genes by lipopolysaccharides in human alveolar macrophages [82], and we identified USP41-specific peptides by mass spectrometry in HEK-293T cells [80]. Whether USP41 is a pseudogene or not, its CD displays a remarkable 97% identity with USP18’s CD, and yet, USP41 appears inert toward ISG15. By combining several biochemical assays, we defined two new features explaining the basis for this difference in enzymatic activity. We identified a stretch of six conserved residues on USP18 C-terminus, forming the amino acid sequence ETAYLL that we named the ‘TAIL motif’ (Figure 3A). We found that Tyr363 (Tyr358 in mUSP18/Ubp43) from the TAIL motif is a key residue which mediates the interaction with ISG15, likely by engaging Arg153 of hISG15 (Arg151 in mISG15, Figure 3B). Accordingly, deletion of the TAIL motif or mutation of Tyr363 rendered USP18 catalytically inactive *in vitro* since it abrogated binding to ISG15. Importantly, while this TAIL motif is not present on USP41, its addition did not, on its own, confer DIG activity to USP41. This suggested the necessity for additional features which are critical for DIG activity. Additional comparative sequence analysis coupled with mutagenesis and enzymatic assays identified USP18 Leucine 198 as critical to mediating USP18’s enzymatic function [80].

While it is generally accepted that USP18 represents the key DIG in cells [83], additional proteases can hydrolyze ISG15. Such proteases were found by using ISG15-specific activity-based probes (ABPs), which contain a reactive group, usually an electrophile, often referred to as a ‘warhead’. The warhead can be a vinyl-sulfone (VS), vinyl methyl ester (VME), or propargylamide (PA) group appended to the C-terminus of ISG15 [84]. Since most DUBs are cysteine-based proteases with active site nucleophiles, these enzymes are well suited for analysis with ABPs [85]. Indeed, the warhead appended to ISG15 will form a covalent bond with the DIG, or DUB, active site cysteine following nucleophilic attack. Thus, covalent attachment of an ISG15-ABP reveals the ability of a DUB to cross-react with ISG15, which we name DIG/DUB. These probes were developed by the Ploegh lab, who used them to screen for DIG/DUBs [86,87]. By combining ubiquitin- and ISG15-ABPs (Ub-VME or ISG15-VS, respectively) with an *in vitro* reconstitution system, USP2, USP5, USP13, and USP14 were identified as DIG/DUBs [88], even though the physiological function of these cross-reactivities still remains unclear.

Three additional studies revealed USP21, USP16, and USP24 as DIG/DUBs [89–91]. The Komander lab reported that USP21, a broadly non-specific DUB which efficiently hydrolyzes all ubiquitin linkages *in vitro* and a paralog of USP2, is a DIG/DUB [89]. By solving the structure of USP21 in complex with a linear diUb aldehyde, a second Ub-binding surface on the USP21 catalytic core was identified. Using biochemistry and enzymatic assays, it was found that USP21 also reacts with ISG15. This was explained by the presence of that second Ub-binding surface, necessary for diUb recognition, since ISG15 is, in essence, a Ubl that resembles a linear diUb (Figure 1B). Consistently, USP21 could remove ISG15 *in vitro* from a cellular lysate of interferon-treated HeLa cells, in a manner dependent on that second Ub-binding site. However, the importance of USP21’s DIG activity during interferon response, as well as the identity of potential substrates, remains unknown.

The other two studies combined ISG15-ABPs with mass spectrometry to identify DIG/DUBs [90,91]. First, an ISG15-ABP containing a biotin affinity tag was incubated with HAP1 cellular lysates, and proteases that reacted with the probe were recovered by streptavidin pull down. Further analysis by LC-MS/MS identified four DIG/DUBs: USP18, USP14, USP5, and USP16 [90]. USP16 has been previously linked to the ubiquitination of H2A, RPS27a, JAK1, and IKKβ [92]. Biochemical assays showed that recombinant USP16 cleaved pro-ISG15 and ISG15 isopeptide-linked model substrates *in vitro*. Finally, to identify ISGylated substrates of USP16, the authors performed an ISG15 interactome analysis by combining ISG15 immunoprecipitation with mass spectrometry, in WT or USP16 knockout HAP1 cells treated with interferon. This revealed that USP16 controls the ISGylation status of metabolic enzymes, such as GOT1, ALDOA, SOD1, and MDH1, consistent with a role for ISG15 in regulating cellular metabolism and mitochondrial function [90]. Interestingly, a truncated version of USP16 containing only its CD retained DIG activity, so the molecular basis for USP16 cross-reactivity with ISG15 remains to be determined. It will be interesting to compare the structures of USP16 CD with either ubiquitin or ISG15 to understand what residues are important in mediating recognition.

Next, a similar study combining biotin-linked ISG15-ABP with lysates of EL4 mouse cells identified USP24 as another DIG/DUB [91]. This study also recovered USP5, USP14, and USP16, corroborating prior reports, while the absence of USP18 could be explained by lack of interferon treatment prior to lysate preparation. This report demonstrated that USP24 can react with mouse and human ISG15-ABPs, in addition to ubiquitin, establishing USP24 as a DIG/DUB. To define the USP24-dependent ISGylome, USP24 was depleted using RNAi in cells treated with interferon beta, and cell lysates were treated with the ubiquitin-selective DUB USP2 to remove ubiquitin from substrates. The resulting samples were digested with trypsin, leaving a di-glycine remnant (GG) only on lysine residues that had been modified with ISG15. By using a di-Gly antibody recognizing Lys-ε-Gly-Gly remnants on proteins after trypsinization [93], peptides containing ISG15 remnants were precipitated, and LC-MS/MS was used to identify the modified proteins. Among these, the RNA helicase MOV10, which is involved in the regulation of gene expression, was shown to undergo USP24-dependent deISGylation, which is important for negatively regulating interferon production and secretion [91]. Notably, USP24 is one of the largest members of the USP family, with an amino acid length of 2620 residues. Strikingly, only full-length USP24 could process ISG15, but not its isolated CD. This demonstrates that the specificity of USPs for their Ubl substrate can be encoded both within and outside the core CD. This is notable since many UBL deconjugating enzymes are studied *in vitro* using only their CDs, which could lead to an incomplete understanding of their activities.

## Alterations of protein ISGylation by viruses

Given the importance of ISGylation during infection, many viruses encode proteases that cleave ISG15 from viral and host proteins to hijack its antiviral function [94]. This is best exemplified by coronaviruses, which target ISGylation to antagonize the host immune response [95,96] (Figure 2, right). Coronaviruses include SARS-CoV-2, the biological agent responsible for the COVID-19 pandemic, as well as SARS-CoV and MERS-CoV, responsible for the Asian 2002 and Middle East 2012 outbreaks, respectively [97]. While the severity of these diseases differs, they all cause serious respiratory illnesses, with symptoms including shortness of breath, fever, and dry cough. These pathogenic effects and the COVID-19 pandemic spurred efforts to characterize the viral life cycle. The prototypical coronaviral genome encodes either the subgenomic RNAs (sgRNAs) or ORF1a/b [98]. The sgRNAs directly produce the structural and accessory proteins of coronaviruses such as the spike (S), membrane (M), envelope (E), and nucleocapsid (N) proteins. In contrast, ORF1a/b will produce 2 precursor polypeptides called pp1a and pp1ab, which will undergo proteolytic cleavage to produce 16 non-structural proteins (Nsps) [98]. Coronaviruses rely on 3-chymotrypsin-like protease (3CLpro, also called the main protease) and papain-like protease (PLpro) to process these precursor polypeptides and facilitate virus replication [99]. 3CLpro, encoded by the nsp5 gene, cleaves pp1a at 11 conserved sites to generate 12 active Nsps (Nsp4–Nsp16). PLpro, encoded by the nsp3 gene, cleaves pp1a at 3 conserved sites to generate Nsp1, Nsp2, and Nsp3 (Figure 2, right). An interesting feature of PLpro lies within the cleavage site between these different Nsps, (R/K)LXGG↓XX, which is similar to the LRLRGG C-term sequence of ubiquitin and ISG15, necessary for covalent attachment to substrates. Thus, PLpro is a viral DIG/DUB that cleaves host ubiquitin and ISG15. Since PLpros and human DUBs share a common mechanism of action, structural studies have highlighted their similar architecture and the common organization of their catalytic cores [80] (Figure 3A and C). The enzymatic activity of both PLpros and USP-family DUBs depends on the same catalytic triad of Cis-His-Asp/Asn residues typical of cysteine proteases [11]. Remarkably, these residues overlay almost perfectly between PLpro and USPs [80], suggesting that they originate from a common ancestor and could be considered part of the same protease family. The main difference between USPs and PLpros is the existence of a Ubl domain in PLpros (Figure 2), whose function is still poorly understood [100]. These structural studies also highlighted how PLpros differ from USP18 in their interaction with ISG15. While USP18 recognizes the C-terminal Ubl domain of ISG15, leaving the N-terminal domain free [78], PLpros interacts with both [100] (Figure 3C, D and E). This has been attributed to their dual DIG/DUB activity, which could enable distinction between ISG15 and monoUb or diUb modifications [100]. Finally, even though PLpros from different coronaviruses are DIG/DUBs, they display preferences over one or the other (Table 1). PLpro from SARS-CoV-2 preferentially cleaves ISG15 from substrates, whereas PLpro from SARS-CoV prefers polyubiquitin chains [102]. Understanding the basis for such preferences and how they relate to immune-escape strategies for pathogens represents important questions that are still being worked on.

**Table 1:** Selected examples of viral proteases possessing DIG and/or DUB activity, including their classification, name, family, cross-reactivity, and preference toward ISG15 or Ub.

Viral DIG/DUBs (non-exhaustive)				
Virus	Classification	Protein name	DUB family	Ubl activity and preference
*SARS-CoV*	Coronaviridae	PLpro	USP	Ub>ISG15 [99,101]
*MERS-CoV*	Coronaviridae	PLpro	USP	ISG15>Ub [99,101]
*SARS-CoV-2*	Coronaviridae	PLpro	USP	ISG15>Ub [99,102,103]
*CCHFV*	Nairoviridae	vOTU	OTU	Ub=ISG15 [104]
*PRRSV*	Arteriviridae	PLP2 (NSP2)	OTU	Ub>ISG15 [105,106]
*FMDV*	Picornaviridae	Lb^pro^	USP	ISG15 (irreversible) [107]

Note that PLP2 from PRRSV is encoded by the NSP2 domain.

**Table 2:** List of human DIG/DUBs including their family, cross-reactivity, and substrates.

Human DIG/DUBs			
Protein name	DUB family	Ubl activity	ISG15 substrates
USP18	USP	ISG15[79], Ub [70–76]	Many [57,124], including STAT1 [125], MDA5 [126], IRF3 [127], RIG-1 [128], or PKR [129]
USP5	USP	Ub[130], ISG15 [85,88]	Unknown
USP14	USP	Ub[131], ISG15 [88]	Unknown
USP21	USP	Ub[132], ISG15 [89]	Unknown
USP16	USP	Ub[92], ISG15 [90], Fubi [133]	MDH1, GOT1, ALDOA, SOD1 [90]
USP24	USP	Ub [134], ISG15 [91]	MOV10 [91]

Other viruses use a viral OTU (vOTU) protease to antagonize ISGylation of host or viral proteins [108]. As their name implies, they are related to the human OTUs (hOTUs), the second largest DUB family after the USPs. hOTUs can hydrolyze certain types of ubiquitin chains [109], and vOTUs also share this feature [110,111]. However, these vOTUs are also DIG/DUBs, such as the vOTU from the Crimean-Congo hemorrhagic fever virus (CCHFV). CCHFV is a deadly human pathogen from the *Nairoviradae* family, whose symptoms include fever, muscle pain, headache, vomiting, diarrhea, bleeding into the skin, and complications such as liver failure [112]. It is encoded by three single-stranded negative-sense RNA segments referred to as the small (S), medium (M), and large (L) segments [113]. The multifunctional L polyprotein, produced by the L segment, encodes the RNA-dependent RNA polymerase, as well as a vOTU at its N-terminus [114]. The CCHFV vOTU cleaves ISG15 with similar kinetics as ubiquitin, and crystal structures of vOTU in complex with Ub or ISG15 provided a molecular understanding of this cross-reactivity [104]. An extension in the N-terminus of vOTU binds the ubiquitin hydrophobic Ile44 patch, resulting in a different ubiquitin orientation compared with a hOTU:Ub complex. Importantly, the C-terminal Ubl fold of ISG15 adopts a similar orientation, and interestingly, that C-terminal lobe of ISG15 contains an additional second hydrophobic surface that is specifically contacted by vOTU.

The Porcine reproductive and respiratory syndrome virus (PRRSV) from the *Arteriviridae* family also encodes another vOTU DIG/DUB. PRRSV is a swine pathogen, whose infections lead to reproductive impairment or failure in breeding animals, as well as respiratory disease in pigs of any age [115]. PRRSV strains contain at least 11 ORFs, with ORF1a and ORF1b representing ~75% of its viral genome. They generate two precursor polypeptides, pp1a and pp1ab, which are processed into at least 16 Nsps. Among them, PRRSV Nsp2 is liberated from the precursor via cleavage of the nsp2:nsp3 junction by the protease activity of Nsp2 [116]. This enzymatic activity lies in the Nsp2 N-terminal region, which encodes a papain-like cysteine protease domain (PLP2) that cleaves a conserved GG dipeptide between Nsp2 and Nsp3 [117]. Bioinformatics analysis found that PLP2 is related to the OTU DUBs [118], with some isolates displaying up to 80.5% sequence identity with their human counterparts. PRRSV PLP2 interferes with K63-Ub chain signaling pathways [105,119], which are involved in regulating the innate immune response through induction of interferon [120]. Moreover, it was suggested that PLP2 could also target ISG15 [105], and biochemical analysis from highly pathogenic, moderately virulent, low virulent, or vaccine derivative strains of Type 2 (North American) PRRSV compared the enzymatic activities of their respective PLP2 [106]. It was found that their enzymatic activity for K63-Ub chains was correlated with the virulence of the strains, i.e. the more virulent PRRSV is, the more active PLP2 becomes. Interestingly, PLP2 from a low virulence strain displayed a noticeably higher DIG activity compared with other strains, thus explaining differences in enzymatic specificities observed in earlier studies. By comparing the available biochemical data of PLP2 from different strains, it was found that two amino acids, E139 and F147, are particularly important for DIG activity, thus providing important insight into the molecular determinants of PRRSV PLP2 substrate preference.

Finally, the cow pathogen foot-and-mouth disease virus (FMDV) from the *Picornaviridae* family also antagonizes ISG15 [107]. FMDV encodes 14 mature proteins, including the leader papain-like protease Lb^pro^, which releases itself from the precursor polypeptide by cleaving its own C-terminus. Lb^pro^ contributes to viral replication by cleaving the two isoforms of the eukaryotic initiation factor 4G (eIF4G) [121], which will divert ribosomes to preferentially translate viral RNA using their internal ribosome entry site. Moreover, Lb^pro^ will cleave ISG15 in an irreversible way. All the proteases discussed so far remove ISG15 by cleaving the isopeptide bond between ISG15 C-term diGly and the lysine residue on the substrate. Through structural and biochemical studies, the Komander lab reported that Lb^pro^ uniquely cleaves a peptide bond in ISG15’s C-terminus, right before the diGly motif, resulting in ISG15 removal from proteins, but leaving lysine residues modified with a diGly remnant. This mechanism of incomplete cleavage irreversibly inactivates ISG15, which can no longer be conjugated. And since the substrate is left with a diGly remnant on the former site of conjugation, this precludes its remodification by another modifier. This unique feature of Lb^pro^ highlights its crucial role in contributing to viral replication through different molecular mechanisms [122]. Whether other viruses and pathogens use a similar strategy to irreversibly inactivate ISG15 still remains unknown.

## Concluding remarks

In summary, the conjugation of ISG15 is a crucial defense mechanism of our immune system to combat viral and bacterial infections, and an important mediator of interferon signaling (Figure 1C). Interferons are molecules that have beneficial properties, but when persistent, they are associated with various human pathologies [123]. Therefore, as an effector of interferon signaling, ISG15 needs to be deconjugated, and this is primarily achieved by USP18, as well as USP16 and USP24 in humans, whose DIG/DUB activity contributes to tissue homeostasis by antagonizing interferon signaling through ISG15 deconjugation. Given the importance of ISG15 as an antiviral agent, several viruses encode proteases that present DIG/DUB features and allow viral replication by antagonizing ISGylation (Figure 2). Thus, the enzymatic functions of these human and viral proteases make them fascinating molecules that are critical to understand at a molecular level. On one hand, the study of human DIG/DUBs has the potential to help us understand the regulation of interferon signaling in greater detail (Table 2). On the other hand, characterizing the mode of action of viral DIG/DUBs could guide the design of pharmacological inhibitors to stop viral replication [99] (Table 1). This is best exemplified by current efforts to find inhibitors of the DIG/DUB PLpro from SARS-CoV-2 [135], the virus responsible for COVID-19, which is crucial for the spread of the disease. Thus, understanding DIG/DUB mechanisms has the potential to find safe and efficient drugs for the treatment of various pathogenic conditions.

**Perspectives**

- Interferon-stimulated gene 15 (ISG15) is one of the strongest interferon-stimulated genes following pathogenic infection, and ISGylation plays a key role in our innate immune system. The importance of ISG15 as an antiviral agent is illustrated by the existence of viral proteases which hijack ISGylation.
- However, deISGylation is equally important to avoid chronic inflammation, or even cell death, and this is mostly achieved by the human deISGylating enzyme (DIG) ubiquitin-specific protease 18 (USP18). Moreover, premature deISGylation by viral DIG/deubiquitinating enzymes (DUBs), such as PLpros from coronaviruses, facilitates viral replication.
- As cysteine proteases, one of the most exciting aspects of DIG/DUBs is their potential as drug targets. Whether small-molecule inhibitors of USP18 or PLpros could enhance the antiviral response of ISG15, with limited side effects, is an important area of future research.

## Abbreviations

ARIH1: Ariadne homologue 1
CCHFV: Crimean-Congo hemorrhagic fever virus
DIGs: deISGylating enzymes
DUBs: deubiquitinating enzymes
E1: ubiquitin-activating enzyme
E2: ubiquitin-conjugating enzyme
E3: ubiquitin ligase
E3: ubiquitin ligase
FMDV: foot-and-mouth disease virus
IFN: Interferon
ISG15: Interferon-stimulated gene 15
JAKs: Janus activated kinases
OTUs: ovarian tumor proteases
PLpro: papain-like protease
PRRSV: Porcine reproductive and respiratory syndrome virus
PTMs: Post-translational modifications
STAT: signal transducer and activator of transcription
TRIM25: tripartite motif
USPs: ubiquitin-specific proteases
Ubl: Ubiquitin-like
VME: vinyl methyl ester
VS: vinyl-sulfone
hOTUs: human OTUs
mISG15: mouse ISG15
vOTU: viral OTU
